# Intestinal Malrotation Through Acute Obstructive Abdomen in an Adolescent From Northeastern Brazil: A Case Report

**DOI:** 10.1155/carm/5628089

**Published:** 2026-04-17

**Authors:** Daniel Vieira de Oliveira, Pedro Melo Toledo Nascimento, Rita de Cassia Almeida Vieira

**Affiliations:** ^1^ Department of Medicine, Universidade Federal de Sergipe, Lagarto, Sergipe, Brazil, ufs.br; ^2^ Department of Nursing, Universidade Federal de Sergipe, Lagarto, Sergipe, Brazil, ufs.br

**Keywords:** Braun anastomosis, gastrointestinal surgery, outcome, volvulus of midgut

## Abstract

Intestinal malrotation is a congenital anomaly of the midgut resulting from abnormal embryonic rotation. Although predominantly a neonatal diagnosis, it is an extremely rare, isolated finding in adolescents, often presenting with nonspecific symptoms that lead to diagnostic delays and increased morbidity. We report a 17‐year‐old male presenting with acute abdominal pain, distension, and vomiting. Diagnosis was established via contrast‐enhanced computed tomography (CT), which identified spiral rotation of mesenteric vessels and colonic displacement. The patient initially underwent a laparoscopic Ladd’s procedure; however, postoperative complications involving duodenal stenosis and recurrent obstruction required a conversion to a Billroth II gastrojejunostomy with a Braun anastomosis. This case highlights that while the Ladd’s procedure remains the surgical standard, the management of malrotation in older patients presents unique challenges compared to neonates. Unlike pediatric cases often associated with other malformations, adolescent presentation is typically isolated and insidious, making contrast‐enhanced CT essential for identifying the “whirlpool sign” and avoiding misdiagnosis. Furthermore, this report underscores that chronic inflammation or recurrence in adults may demand surgical strategies beyond the standard procedure. In complex scenarios with recurrent obstruction, reconstructive techniques such as the Billroth II gastrojejunostomy with Braun anastomosis or Roux‐en‐Y gastric bypass are effective alternatives to prevent alkaline gastritis and ensure long‐term symptom resolution.

## 1. Introduction

Intestinal malrotation is a congenital anomaly resulting from the abnormal rotation and fixation of the midgut during fetal development [1, 2]. It is frequently diagnosed in neonates, often associated with other defects like diaphragmatic hernia [3]. However, malrotation is extremely rare in adolescents and adults. In this older demographic, it typically presents as an isolated finding [4].

Clinical presentation in adolescents is often insidious. Patients report nonspecific symptoms, including chronic abdominal pain, nausea, vomiting, and bloating. These vague complaints mimic other gastrointestinal disorders, leading to diagnostic delays [5]. Consequently, accurate imaging is vital. While abdominal radiography and ultrasound are useful, contrast‐enhanced computed tomography (CT) and upper gastrointestinal (UGI) series serve as the diagnostic gold standards [6–8].

**FIGURE 1:**
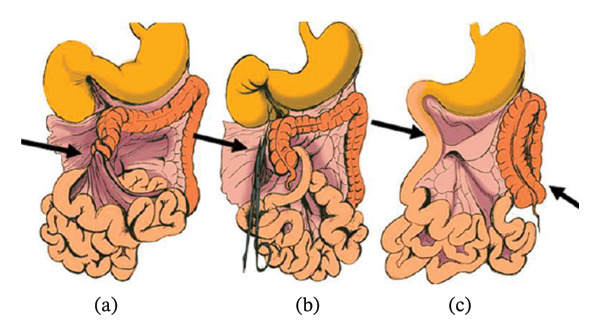
Ladd’s procedure [9]. Picture from Biko et al. [9].

Once diagnosed, the definitive treatment is the Ladd’s procedure (Figure 1). This surgery releases fibrous bands and straightens the mesentery [9]. It can be performed safely via laparoscopy or open laparotomy. Both approaches are effective for symptom resolution [10, 11]. Timely intervention and postoperative monitoring are crucial to prevent complications such as midgut volvulus and ischemia [12, 13].

Herein, we describe a challenging case of recurrent duodenal obstruction in a 17‐year‐old patient following a Ladd’s procedure, ultimately requiring a Billroth II gastrojejunostomy.

## 2. Case Report

A previously healthy 17‐year‐old male was admitted to the emergency room complaining of moderately intense, colicky abdominal pain in the left quadrants, associated with nausea and vomiting. On physical examination, the abdomen was soft with mild diffuse tenderness, most notable in the left hemiabdomen, but without peritoneal irritation. An initial noncontrast CT scan ruled out obvious obstruction or volvulus but revealed enlarged mesenteric lymph nodes. Although the radiologist noted the possibility of internal hernia or malrotation, the attending physician considered mesenteric adenitis as the primary hypothesis due to the lymphadenopathy. Retrospectively, it is acknowledged that a detailed history regarding chronic symptoms was not obtained during this acute presentation; such an inquiry might have strengthened the suspicion of malrotation. Consequently, the patient was discharged with analgesics and ciprofloxacin for empirical treatment of the presumed infectious etiology.

Upon return 2 days later, the patient reported worsening abdominal pain. A detailed anamnesis revealed a history of recurrent, severe abdominal pain associated with physical exertion over several years, with intermittent exacerbations. On physical examination, the abdomen exhibited slight distension and intense tenderness on palpation, though signs of peritoneal irritation were absent. Cardiopulmonary auscultation was normal, and screening for associated congenital anomalies yielded negative results.

**FIGURE 2:**
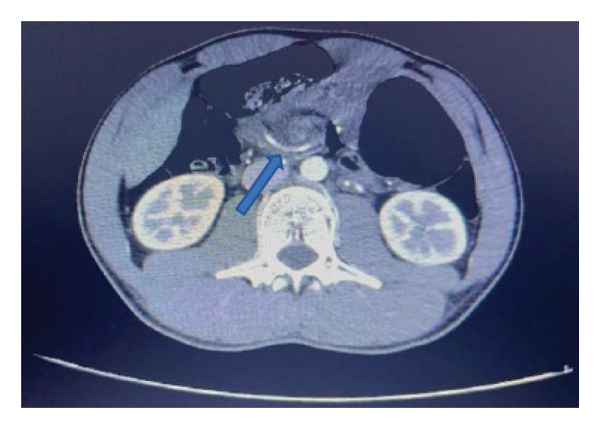
Computed tomography scan showing spiral rotation of the mesenteric vessels.

**FIGURE 3:**
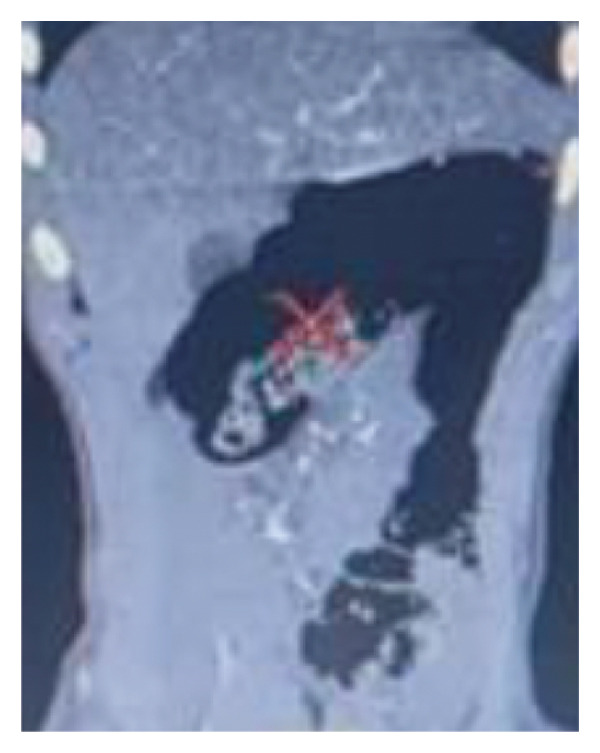
Computed tomography scan showing abnormal positioning of the cecum and distension of colonic loops.

A new CT scan with intravenous contrast was performed, suggesting intestinal malrotation. It revealed spiral rotation of the mesenteric vessels (Figure 2), abnormal cecal positioning, distension of colonic loops (Figure 3), and mesenteric vein distension with superior mesenteric artery compression, without signs of bowel wall thickening. On the same day, he underwent exploratory video‐laparoscopy, confirming incomplete malrotation with the cecum located horizontally in the upper abdominal midline. A laparoscopic Ladd’s procedure was performed, including cecoduodenal adhesiolysis, small bowel repositioning, and appendectomy, without complications. The patient recovered well postoperatively, tolerating diet progression and passing stool. He was discharged on postoperative day (POD) 2 without symptoms of obstruction.

Seventeen days after discharge, the patient went to another medical service with abdominal pain and mild dietary intolerance. Over the preceding 3 days, he could only tolerate liquids and soft foods. Another CT scan revealed gastroduodenal distension, suggesting potential duodenal obstruction.

A second video‐laparoscopy was performed on the same day. The procedure involved rectifying the duodenal transition by correcting the torsion and dissecting adhesions in the third and fourth portions of the duodenum. The operation was uneventful, and the patient recovered well until POD 6.

On POD 6, the patient experienced vomiting and absence of bowel movements. A CT scan with oral and intravenous contrast indicated a caliber constriction in the fourth portion of the duodenum. Consequently, the patient was started on total parenteral nutrition (TPN) on POD 7, and surgical planning was initiated. A repeat CT scan on POD 9 confirmed persistent gastric distension with duodenal narrowing. On POD 10, the patient underwent an exploratory laparotomy. The decision to proceed with an open approach was driven by the anticipated technical difficulty. Which was led by a board‐certified general and gastrointestinal surgeon with over 15 years of experience, safe mobilization and dissection of the third and fourth duodenal portions are extremely challenging via laparoscopy, particularly in a reoperative field with dense inflammatory adhesions. The laparotomy revealed stenosis in these segments. A duodenojejunostomy was initially considered; however, attempting a direct anastomosis in this inflamed, friable tissue was deemed to carry a high risk of leakage. Therefore, the team opted for a Billroth II gastrojejunostomy to bypass the hostile duodenal environment entirely, ensuring effective drainage. A Braun anastomosis was added specifically to prevent alkaline reflux gastritis.

Postoperatively, the patient remained stable. On POD 3 after the third surgery, a liquid diet was initiated via a nasoenteric tube and was well tolerated. Although initially apprehensive about resuming oral intake due to fear of vomiting, the patient was reassured regarding the gradual dietary progression. In the subsequent days, he improved, accepting a cautiously advanced oral diet and successfully discontinuing TPN.

The patient was discharged on POD 8, tolerating a mild oral diet with normal bowel movements and an unremarkable abdominal examination. Postdischarge, he resumed daily activities and reported a favorable clinical course during follow‐up, with no new complaints.

**FIGURE 4:**
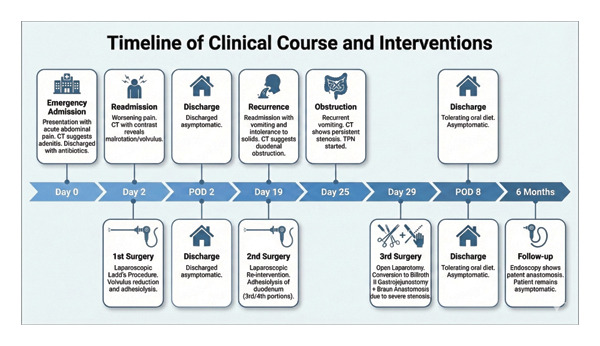
Timeline of the clinical course, surgical interventions, and outcomes. Legend: CT—computed tomography; POD—postoperative day; TPN—total parenteral nutrition.

**FIGURE 5:**
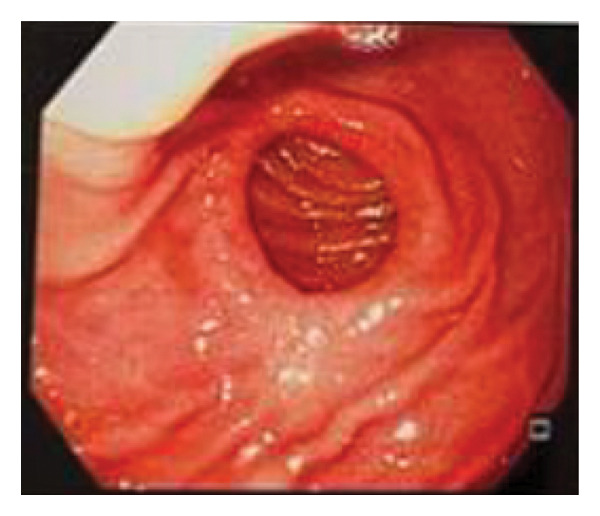
Esophagogastroduodenoscopy shows pervious gastrointestinal anastomosis.

**FIGURE 6:**
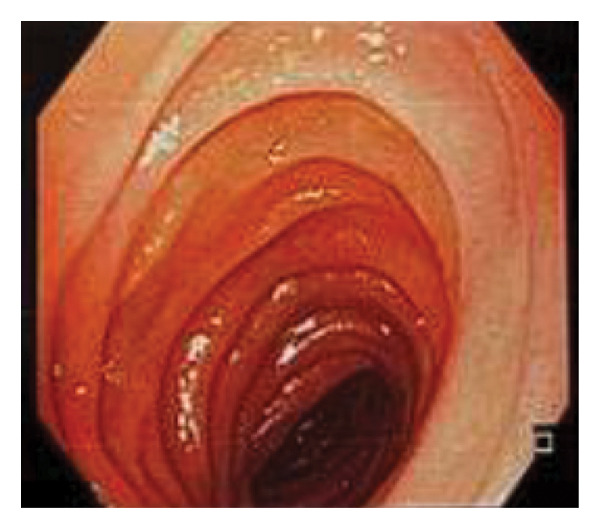
Esophagogastroduodenoscopy shows a patent afferent jejunal loop.

**FIGURE 7:**
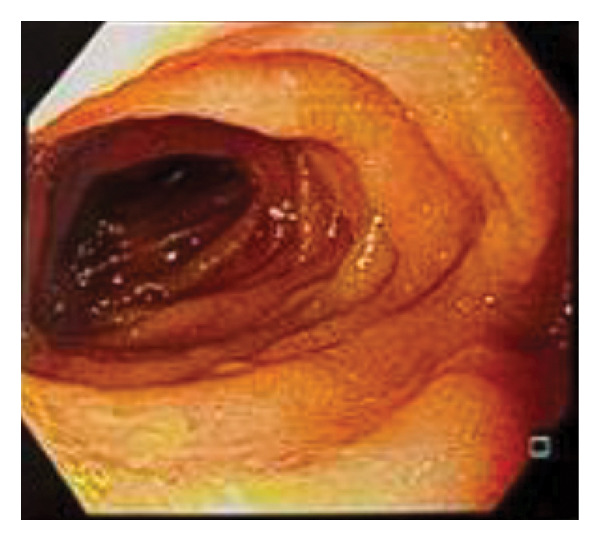
Esophagogastroduodenoscopy showing an efferent jejunal loop with biliary content.

Six months after the final surgery, a follow‐up esophagogastroduodenoscopy (EGD) was performed (see Figure 4). The examination revealed a patent gastrojejunostomy, indicative of a successful Billroth II reconstruction. Notably, the EGD showed a fully distensible stomach without signs of stenosis or alkaline gastritis (Figure 5) in the afferent (Figure 6) or efferent limbs (Figure 7). No abnormalities were observed.

## 3. Discussion

Intestinal malrotation is a congenital anomaly of the gastrointestinal tract resulting from the failure of normal midgut rotation during embryonic development [2]. While frequently associated with other congenital defects in neonates (such as diaphragmatic hernia or omphalocele) [14–20], malrotation in adults is an extremely rare, isolated finding, with an estimated incidence of 0.2%–0.5% [21]. Unlike the acute presentation in infants, adult patients typically exhibit chronic, insidious, and nonspecific symptoms, including abdominal pain, vomiting, and dietary intolerance [8].

Due to this symptom nonspecificity and the condition’s rarity in adulthood, malrotation is seldom a primary diagnostic hypothesis. Consequently, patients often experience significant diagnostic delays, as observed in the present case [22]. Such delays are clinically significant because they are linked to increased morbidity, as illustrated by our patient who was diagnosed only upon presenting with acute volvulus and obstruction [5, 7, 8, 12, 23].

Given these diagnostic challenges, accurate imaging becomes essential. While UGI series are the gold standard for pediatric patients [6], contrast‐enhanced CT is the preferred modality for adults. CT can identify characteristic findings such as the ‘whirlpool sign’ and anomalous colonic positioning [1, 5]—both of which are evident in our patient. Radiography and ultrasound generally offer limited utility in this demographic [5, 8].

Regarding treatment, the standard of care is the Ladd’s procedure, which entails volvulus reduction, band lysis, appendectomy, and mesenteric repositioning [10, 24]. We initially opted for a laparoscopic approach, which is well‐supported in the literature for having outcomes comparable to open surgery [24, 25]. However, the patient developed a recurrent obstruction requiring re‐intervention—a known risk particularly in older children and adults due to chronic inflammatory changes [4, 13].

Faced with this recurrence, a strategic change in surgical tactic was necessary. Aligning with Raitio and colleagues, who suggest alternative reconstruction techniques for recurrences in older pediatric patients [4], we performed a Billroth II gastrojejunostomy. Furthermore, a Braun anastomosis was added to specifically prevent alkaline reflux gastritis and minimize the risk of future adhesive episodes [26].

This case underscores the inherent complexity of intestinal malrotation when it presents outside the neonatal period. The combination of nonspecific symptoms and delayed presentation significantly increases the risk of severe complications. Highlighting the uniqueness of this challenging scenario, only 11 cases of concurrent intestinal malrotation and duodenal stenosis have been reported in the literature to date [15–19, 21, 27–31].

Ultimately, managing this condition in older demographics demands clear clinical strategies. First, a high index of suspicion is required when evaluating adult or adolescent patients with chronic or recurrent abdominal symptoms to avoid critical delays. Second, contrast‐enhanced CT must be utilized as the gold standard for diagnosis, enabling the early identification of anatomical anomalies. Finally, while the Ladd’s procedure remains the first‐line surgical treatment, operative teams must be prepared for complex intraoperative environments. In cases where severe chronic inflammation and dense adhesions preclude a standard outcome or lead to recurrent obstruction, alternative reconstruction techniques—such as a Billroth II gastrojejunostomy—may be necessary to ensure a safe and definitive resolution.

## 4. Strengths and Limitations of This Report

The primary strength of this case report lies in the detailed documentation of a rare, challenging complication—severe inflammatory duodenal stenosis following a Ladd’s procedure in an adolescent—and the successful application of a Billroth II gastrojejunostomy as a salvage technique. The comprehensive clinical timeline and endoscopic follow‐up further strengthen the reliability of our findings. However, this study has inherent limitations. As a single retrospective case report, the findings cannot be broadly generalized. The initial delay in diagnosis, while reflecting real‐world clinical challenges in emergency settings, also highlights the need for larger multicenter case series to establish standardized protocols for complex adult and adolescent presentations of malrotation.

## Funding

No funding was received for this manuscript.

## Disclosure

The authors reviewed and edited the content as needed and take full responsibility for the content of the publication.

## Ethics Statement

This study complies with the ethical guidelines of Brazilian legislation and was approved by the Research Ethics Committee (CAAE: 76550423.3.0000.0217). Informed consent was​ obtained from the patient and legal guardians.

## Conflicts of Interest

The authors declare no conflicts of interest.

## Data Availability

The data that support the findings of this study are available on request from the corresponding author. The data are not publicly available due to privacy or ethical restrictions.
